# Colorization and Automated Segmentation of Human T2 MR Brain Images for Characterization of Soft Tissues

**DOI:** 10.1371/journal.pone.0033616

**Published:** 2012-03-27

**Authors:** Muhammad Attique, Ghulam Gilanie, Hafeez-Ullah, Malik S. Mehmood, Muhammad S. Naweed, Masroor Ikram, Javed A. Kamran, Alex Vitkin

**Affiliations:** 1 Department of Computer Science and Information Technology, The Islamia University of Bahawalpur, Bahawalpur, Punjab, Pakistan; 2 Department of Physics and Applied Mathematics, Pakistan Institute of Engineering and Applied Sciences, Nilore, Islamabad, Pakistan; 3 Department of Radiology and Diagnostic Images, Bahawal Victoria Hospital, Bahawalpur, Pakistan; 4 Department of Medical Biophysics and Radiation Oncology, University of Toronto, Toronto, Ontario, Canada; 5 Division of Biophysics and Bioimaging, Ontario Cancer Institute/University Health Network, Toronto, Ontario, Canada; Hangzhou Normal University, China

## Abstract

Characterization of tissues like brain by using magnetic resonance (MR) images and colorization of the gray scale image has been reported in the literature, along with the advantages and drawbacks. Here, we present two independent methods; (i) a novel colorization method to underscore the variability in brain MR images, indicative of the underlying physical density of bio tissue, (ii) a segmentation method (both hard and soft segmentation) to characterize gray brain MR images. The segmented images are then transformed into color using the above-mentioned colorization method, yielding promising results for manual tracing. Our color transformation incorporates the voxel classification by matching the luminance of voxels of the source MR image and provided color image by measuring the distance between them. The segmentation method is based on single-phase clustering for 2D and 3D image segmentation with a new auto centroid selection method, which divides the image into three distinct regions (gray matter (GM), white matter (WM), and cerebrospinal fluid (CSF) using prior anatomical knowledge). Results have been successfully validated on human T2-weighted (T2) brain MR images. The proposed method can be potentially applied to gray-scale images from other imaging modalities, in bringing out additional diagnostic tissue information contained in the colorized image processing approach as described.

## Introduction

Many spectroscopic techniques have been used for diagnostics and assessment of biological tissues, each with their own advantages and drawbacks [1]. In addition to spectroscopy, many approaches also yield spatial maps of tissue structures by recording 2D or 3D image [2], [3], [4], [5]. MR imaging is particularly promising for human brain characterization because of its in-vivo capability, excellent tissue contrast, and high spatial resolution. [6]. Often, however, the obtained images require additional analysis to extract otherwise hidden features, for example image segmentation for visualizing of different brain regions.

Brain MR image segmentation can help identify regions like gray matter (GM), white matter (WM) and cerebrospinal fluid (CSF) for functional visualization in the diagnosis of diseases like stroke and cancer, and neurological disorders like Alzheimer's disease and multiple sclerosis. Drawing on various image analysis/segmentation tools, clinician's ability to perform volume estimation, tissue classification, surface mapping, morphological evaluation, delineation of region of interest (ROI) can be enhanced [6], [7].

Segmentation methods in particular have been actively investigated, generally classified into two groups – hard segmentation divides the image into non-overlapped regions, while soft segmentation allows the regions to overlap, where a single pixel/voxel can be associated with multiple regions [8], [9].

Segmentation of gray scale images can be performed with different methods like thresholding, edge based method, region growth and watershed, and others [8]–[10]. The limitations of these methods include the need for supervision/subjective aspects of method search initialization, high computational cost, and complexity. For example, thresholding approaches do not take consider the spatial characteristics of an image, and the results are prone to the artifacts caused by image noise and inhomogeneities. These essentially corrupt the histogram of the image, making separation of different tissues more difficult [8], [10]. Similarly, in edge based method, noise and intensity inhomogeneities can generate artifactual and weak edges [11], [12]. There is thus a need to explore alternate segmentation methodologies. Specifically, colorized segmentation of gray scale images may yield enhanced results compared to naked eye assessment of normal and diseased tissue based on monochrome data [13], [14].

To avoid subjectivity inherent in operator-supervised segmentation methods, unsupervised clustering methods have been developed that are independent of the training data, instead performing tissue classification tasks iteratively. Initially the centroids (some selected gray levels from the image that can divide the image into some meaningful parts) are randomly selected and revised in each iteration until convergence is reached [7], [8]. However, such random and repetitive iteration can become complex and time consuming. Some of these are K-mean or hard C-mean and fuzzy C-mean clustering methods classify each pixel into distinct clusters nearest to its centroids by measuring the distance between each pixel and the selected centroids using the minimization of objective function [10], [15].

The minimization of objective function is used in a verity of way for data clustering like K nearest neighbor (KNN), Parzen Window, minimum variance quantization (MVQ). KNN algorithm requires the definition of ‘K’ number of neighbors and ‘C’ number of classes. Unlabeled points are then labeled by assigning the weighted majority of their neighbors. The weighted majority is calculated using objective function. The disadvantage with these methods is, the points remain unlabeled if two samples from different classes are closed equally [16]. The Parzen window is a generalized form of KNN, where point labeling is achieved by utilizing the kernel window to estimate the weights of neighbors of that point. Kernel window should be adjusted to some suitable point to obtain the optimum results [16], [17], [18]. The MVQ is based on dithering principal and uses error diffusion for image quantization. It reduces the depth of color by grouping the pixels on the base of their variance [16], [19]. KNN and Parzen window are based on the neighboring point's weight while ignoring the importance of point alone that might be valuable task for pixel classification otherwise. K-mean and Fuzzy C-mean etc are purely pixel classification methods that calculate the centroids iteratively during whole segmentation process that make the computational process more complex [20]. We use the same basic way to implement the minimization of objective function but just introduce a new way to feed this function with auto centroids selection that have to be selected once. This auto centroid selection minimizes the iterative overhead for centroid selection and makes the whole segmentation process a single phase.

Various histogram based segmentation methods have been proposed and available in literature. Panning A et al. [21] proposed an adaptive threshold method for single threshold selection, that initially selects a rough adaptive threshold value from histogram then utilizes Gaussian distribution model to refine the threshold value. In this method there is greater chance of wrong selection of irrelevant pixels. Krstinic D et al. [22] utilized multidimensional color histogram to obtain density estimate based on variable kernel density estimator then these estimated ranges incorporating region growing techniques for image segmentation. This method seems to be efficient enough in obtaining cluster ranges however region growing techniques are somewhat computationally complex. Ilea DE and Whelan PF [15] proposed a multichannel histogram based segmentation method that constructs histogram for red, green and blue color components and then divides each histogram into R regions. This method iteratively selects peaks from each region and constructs new color that is used as centroids. Chaabane SB et al. [23] proposed a method that calculates homogeneity histogram for each color and used the mass estimation function of Dempster Shafer evidence theory on homogeneity histogram for optimal segmentation incorporating fuzzy homogeneity vector. The histogram based methods [24], [25], [26], are mainly used for automatic image thresholding. These methods divide the image in two classes; background and foreground. In some segmentation methods histogram is utilized to calculate membership function for the seed or gradient estimation iteratively while some other incorporates mass/density estimation functions.

Our proposed segmentation method selects the centroids in single iteration for the specific number of regions from the image histogram. For the centroid selection the image histogram is divided into three regions on the base of prior anatomical knowledge that makes centroid calculation phase robust. With this auto centroid selection method we reduced the overhead of the membership function's iterative calculation mostly used with different clustering methods (K mean, Fuzzy C-mean etc). These selected centroids are then used with the minimization of objective function to segment the image.

Different colorization approaches have been defined to acquire color images from the gray images and can be divided into the general and specific categories as per their usage. For grayscale images and videos colorization Jacob VG and Gupta S [27] proposed a semi automatic approach. User interaction is required to choose some reference frames/regions with the desired color marker from the video sequences/image to color. Watershed algorithm is used to segment the image regions for the decision of chosen color shades within the image. Luminance and texture matching approach is used in fully automated colorization of grayscale images using the already constructed image database was proposed by Rathore Y et al. [28]. To construct image data base along with their parameters is the overhead with this colorization approach. Squvaget C et al. [29] suggested completely or partially automatic gray image colorization approach; designed specifically for the illustrators, artists or general users. User can define color from chromatic hue wheel or Itten's proportion contrast based selected harmony can be used to adjust color proportion. Bochko V et al. [30] proposed a colorization method for gray scale medical images using color learning with dichromatic reflection model to predict colors from color image.

Holland GN and Bottomley PA [31] introduced color display technique for NMR images. The famous tool used in scientific labs MATLAB also colors the gray images using its predefined functions. MATLAB supports different types of color-maps (hot, summer and etc) and programmers can also define their own color-map with their own color ranges. It uses linear mapping to assign colors to a gray values. First minimum and maximum values are determined from both the source gray image and from the defined color-map and then minimum color value is assigned to minimum gray value and maximum color value is assigned to maximum gray value of the image. In between the minimum and maximum all other color-map colors are then linearly assigned to each gray value [32]. Different multi-parametric MR images based colorization techniques are also available in the literature. Multi-parametric MR images such as T1 weighted (T1), T2, and FLAIR are used in colorization process and assigns red to T1, blue to T2 and green to FLAIR image of the same anatomical position and then fused them to obtain color composite image [33]. Weiss KL et al. [34] used two MR images of different pulse sequence with same anatomical position and determine the hue and luminance using corresponding pixels of these two images to produce a single colorized image. Similar nature of colorization method is adopted where more than one multi-parametric MR images are used to obtained color image [35], [36].

Whereas in the proposed method of colorization we use a single slice of MR images (either it is T1, T2 or etc) to colorize it by comparing the luminance of each pixel with the provided color image pixel's luminance. We reduce the overhead of the techniques used in [33], [34], [36] where they first encode the separate color to image sequences and then fuse these images to display color data, which is a computationally complex and costly solution. The proposed method retains the original luminance of gray image during colorization process. However the change in luminance may cause artifacts otherwise. Colorization methods used by MATLAB did not take into account the luminance [32]. Another advantage to use single slice colorization method is that it works equally on other medical imaging modalities like CT, OCT, Digital X-Ray and etc where we have only one way to acquire images. We experimentally verified our method on other medical imaging modalities.

In this article, we propose two independent methods; a novel method to colorize the gray scale brain MR images to enhance the visual perception, enabling more precise tissue discrimination, and a customized clustering method for colorized segmentation based on gray images. The clustering process for segmentation is customized by introducing a pilot automatic centroid selection method using prior anatomical knowledge, thus reducing the random (‘blind’) nature of many current clustering methods.

The results indicate that our segmentation method is capable of delineating the brain anatomical structures, yielding more visually probable images. Drawing upon the human visual system's ability to discriminate colors well, the proposed methodology can become useful in enhancing the information content of monochrome MR (and potentially other modality) images.

## Materials and Methods

T2 brain MR images were obtained from total 57 subjects (39 males and 18 females) of average 32 years old normal volunteers and patients having stroke, hemorrhage, tumors and multiple sclerosis. Amongst them 27 subjects (19 males and 08 females) were used to derive criteria for colorization and segmentation process as shown in Table 1. Rest of the 30 subjects (20 males and 10 females) was used to obtained results for the verification of both proposed methods. The images were obtained on a Philips Achieva 1.5 Tesla MRI, with twenty 0.5 mm thick slices for each data set. The experimental work was performed under intuitional laws of Bahawal Victoria Hospital (BVH) Bahawalpur, Pakistan. These laws were validated by institutional review and ethical committees. The participants/patients were informed and signed the consent of data acquisition before measurements in the department of radiology and diagnostic images Bahawal Victoria Hospital Bahawalpur, Pakistan.

**Table 1:** Showing subjects examined for centroid selection and colorization criterias.

Proposed Algorithm	Imaging Modality	Subjects	Normal/Abnormal	Male/Female	Volunteer/Patients	Consultation With Experts & Atlas Used
**Analysis for centroid selection method**	MRI (T2W)	18	13/05	11/07	03/15	Yes [1], [2]
**Analysis for colorization method**	MRI (TW1,T2W, FLAIR)	27	21/06	19/08	05/22	Yes [1], [2]
	CT	10	3/7	4/6	0/10	-
	OCT	10				-

**Figure 1:**
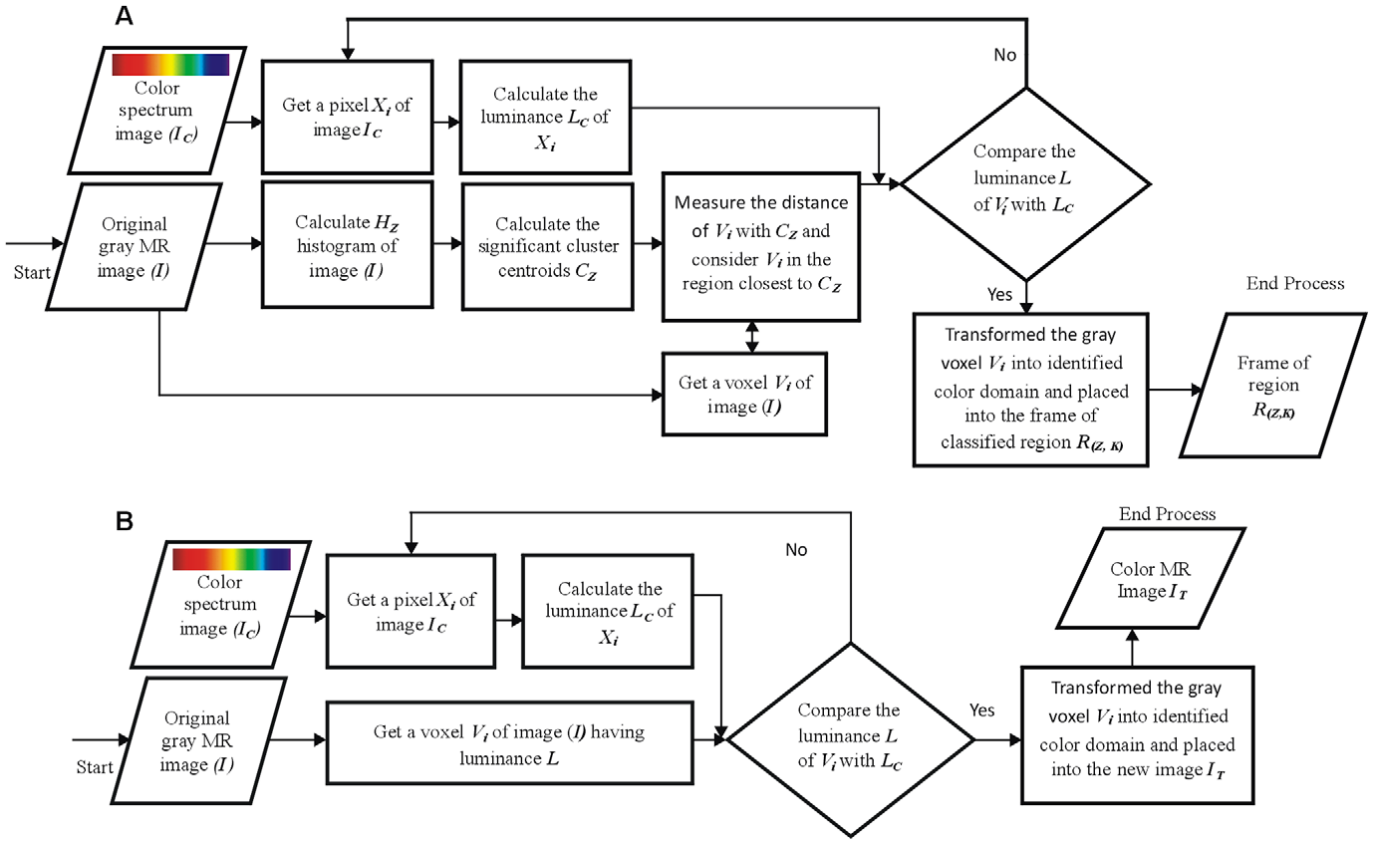
Block diagram of proposed methods. (A) Block diagram of proposed colorization method. (B) Block diagram of proposed segmentation method.

We have developed and used two independent models: a) a colorization method for gray images b) a segmentation method (for both hard and soft segmentation) using a customized single phased clustering method (initialized with auto centroids selection). The colorization and segmentation models are represented with the block diagram shown in Figure 1.

The major regions of T2 brain MR images are Gray Matter (GM), White Matter (WM) and Cerebrospinal Fluid (CSF) to highlight [7], [37]. In this research the prior anatomical knowledge mean is the knowledge of these areas. To get the expert opinion for the study of brain anatomy two senior radiologists of Bahawal Victoria hospital Bahawalpur Dr. Javaid Ahmad Kamran and Mustansar Mehmood Waraich [38], [39] were consulted, who visually guided us by the aid of Philips Dicom Viewer and using atlas's [40], [41], [42]. We visually analyzed the histogram of brain MRI with the consultation of these radiologists and image processing experts and conducted the study to find the significant and representative points for each anatomical region. The literature describes that peaks and valleys represent the object presence in the histogram [43]. Total 27 subjects (19 males and 08 females) were analyzed to derive criteria for both colorization and segmentation process (used in Eqs. (6–7)). Whole set of 27 subjects was used to derive the criterion for colorization process whereas subset of 18 (11 males and 07 females) subjects from these 27 subjects was used to derive the criteria for segmentation process. We conduct our study on the data set described in Table 1 to analyze the variations in the histogram for the peaks and valleys per slice. To segment out these regions we use clustering method that requires the centroids as a seed for each region. Utilizing this prior anatomical knowledge we derived separate region limits and proposed an efficient way to select the appropriate peak points from the histogram of brain MR image. These peak points are centroids (one for each anatomical region).

### Colorization method

To transform a gray scale T2 brain MR into color we have used perceptually uniform CIELAB color space standardized by the Commission Internationale de L'Eclairage (CIE) [13], [44]. A gray image has only luminance or intensity value *L* to represent a pixel with range 0–255 whereas a color image uses RGB color space with *Red*, *Green* and *Blue* correlated components. Therefore, a uniform CIELAB color space has been used to transform the RGB space into the tri stimulus uncorrelated components; luminance component *L* and two chrominance component *a* has red to green affiliation and *b* has blue to yellow affiliation correspondingly. That means the changes in any one of the component has a minor or no effect on the other two components [44], [45].

The basic purpose to use CIELAB is to retain the actual luminance of the original gray image during color transformation. This is achieved by comparing the gray image's each pixel original luminance with the calculated luminance of each pixel of input color spectrum image (range: 400 nm–700 nm). The luminance is calculated using the YIQ (Luma in-phase quadrature) system defined in Eq. [1]. On successful match, chromaticity values are transformed (calculated using CIELAB color space transformation) from the source color spectrum image to the targeted image.

**Figure 2:**
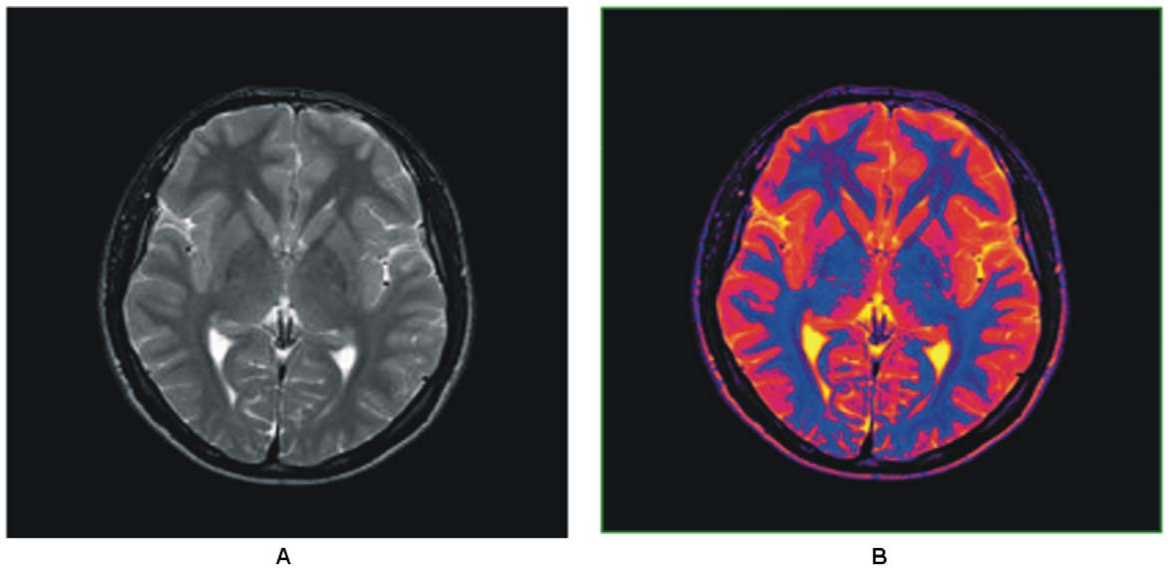
Color transformed T2 brain MR image. (A) Gray scale brain MR image. (B) Colorized brain MR image obtained using proposed method.

The luminescence L of a pixel in RGB spaced was calculated with YIQ system [13] given by eq. (1).
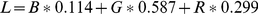
(1)The luminance of a gray scale image pixel contains sufficient information to represent it into its parallel color space and provides extra viscous measures and perceivable variability within the object (local) and in the entire image (global) as shown in Figure 2.

### Steps for colorization method

Suppose “*I*”, “*I_C_*”, and “*I_T_*” *are* input gray image, source color spectrum image and targeted image with the same dimension as of *I*. *P*, *P_C_* and *P_T_* are the pixels of image *I*, *I_C_* and *I_T_* correspondingly.

1. Get a pixel *P* from gray scale image which has only luminance *L*,
2. Get a pixel *P_C_* from source color spectrum image and transform it into the luminance *L_C_* using Eq-1
3. Measure the distance *D* (Euclidean Distance) using Eq. (2) between the luminance values *L* and *L_C_*
  - If the *D* matches the criteria *C_1_* defined below then transform *P* and *P_C_* into CIELAB and assign the luminance *L* and paired chrominance values *a* and *b* of *P_C_* to *P_T_* and then go to the Step-1 until the complete color transformation.
  - Otherwise go to the Step-2
4. Stop the whole process
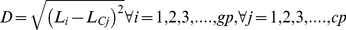
(2)Where 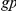 is the total number of gray pixels from T2 brain MR image and 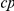 is the total number of color pixels from source color spectrum image. The successful match should follow the criteria 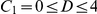. This criterion is proposed after testing on a large set of T2 brain MR images for color transformation shown in Table 1.

Human eye is unable to perceive continuous change in color spectrum. It is identifiable only when there is significant change in color. The criterion range used in colorization process is derived after testing the brain MR data set of 27 normal and abnormal volunteers and patients as shown in Table 1. This criterion is also verified with other medical imaging modalities widely in practiced like CT and OCT. As this criterion efficiently works with various medical imaging modalities, so we introduce this criterion as the best match as it gives appropriate and considerable variations among tissues. There are almost 38 basic soft tissues of human brain described on the behalf of their functionality [40], [41], [42] and to enhance their readability if they are displayed in color we need atleast 38 different human perceivable colors. To achieve this we utilized 200 different human perceivable colors. When this criterion is applied using Euclidean Distance it approximately divides these colors into the bands equal to the number of basic brain soft tissues. Hernandez MDCV et al. [35] experimentally showed that there are 32 color levels sufficient to produced good results for soft brain tissues and proposed a colorization method.

To transform RGB space into CIELAB space tri-stimulus values ‘XYZ’ are calculated by following the way described in [46],[44]. At initial non-linear RGB components are transformed to standard linear 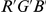 values which can be then transformed into tri-stimulus values ‘XYZ’ to calculate the CIELAB values [44],[47].

### Auto centriod selection model for clustering segmentation

To save time consumption in clustering method due to a large number of iterations for random selection of centriod from image [7], [15] a single phase clustering method with auto selected centroids based on prior anatomical knowledge has been proposed. Simple minimization of objective function is customized for 3D image segmentation as follows
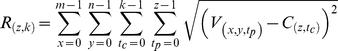
(3)This equation deals 3D images for z>1 and also applicable on 2D images with z = 1. Where 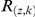 represents the number of identified regions equal to the number of cluster centroids *k* for each image plane *z* and 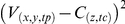 is the distance measure between the voxel 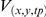 of the T2 brain image and the cluster centroid 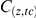. 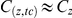, 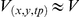 and *n*, *m* are the dimensions of each plane used throughout in this work. Segmentation process accomplishes by using identified centroids *C_Z_* and classifies each voxel *V* into its identical cluster using the objective function described above.

The variable *V(x,y,tp)* denotes an individual voxel at location *(x, y, tp)* that is similar to the coordinates *(x, y,z)* while *C(z,tc)* are the selected centroids with *z* is the number of image slides/planes and *tc* is the total number of centroids (there is separate centroid calculated from each region) for each slide. Overall the variables *(V(x, y, tp) - C(z,tc))* in Eq. (3) represent the difference of gray levels between the voxel *V* at location *(x,y,tp)* from centroid *C(z,tc)*.

**Figure 3:**
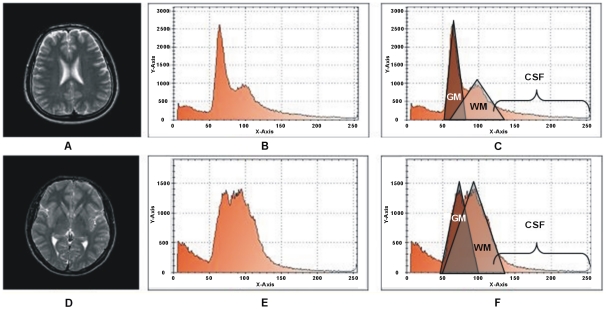
Histogram of T2 brain images with the peak analysis. (A) T2 Brain MR image slice1. (B) Histogram of slice1. (C) Peak analysis based on rectangles drawn. (D) T2 Brain MR image slice2. (E) Histogram of slice2 (F) Peak analysis by based on rectangle drawn.

**Figure 4:**
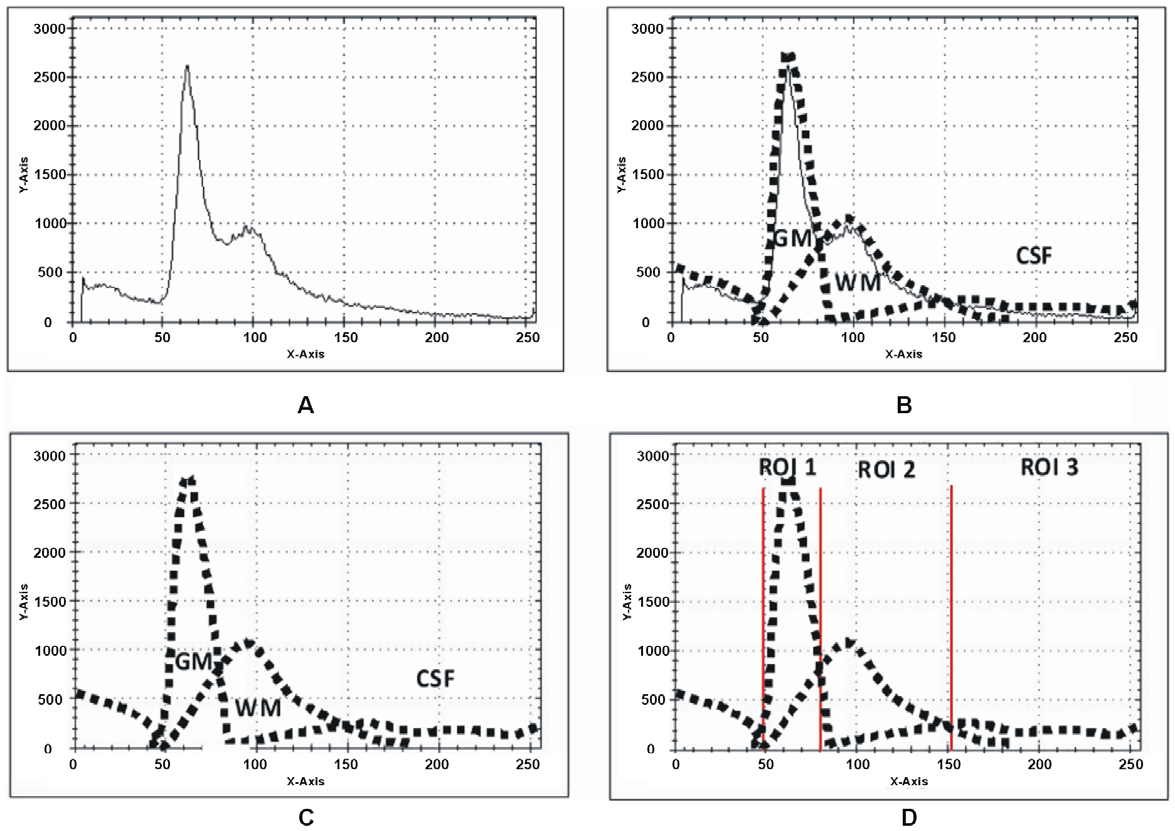
Probabilistic histogram and detailed description of Figure 3. (A) Histogram of Figure 3(A). (B) Lines drawn around the peaks for probabilistic histogram. (C) Probabilistic histogram. (D) Splitting region for ROI at intersection points.

**Figure 5:**
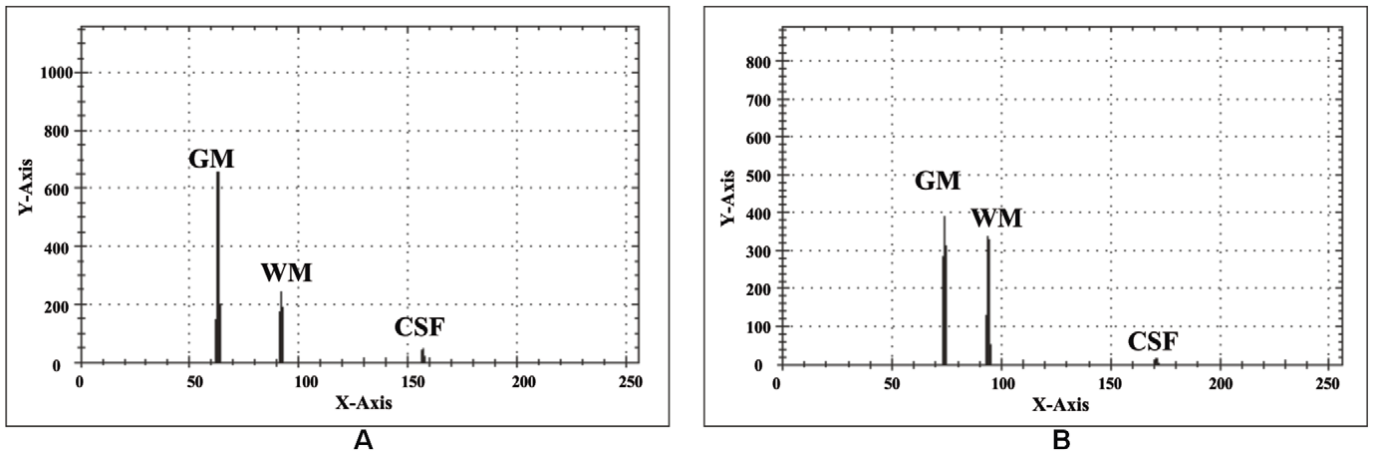
Selected centriods with Auto centriod selection method. (A) Selected centroids within the specified ranges from the histogram shown in Figure 3(B). (B) Selected Centroids within the specified ranges from the histogram shown in Figure 3(E).

For auto centroid selection initially the histogram 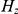 of the input gray image is calculated for each image plane as; 
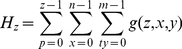
(4)Two different slices of T2 brain MR images with their relative histograms are shown in Figure 3 generated to describe the process of auto centriod selection. Figure 3(A) shows the original gray image of slice 1. Figure 3(B) represents the histogram of slice 1. Figure 3(C) gives the triangle based analysis for GM, WM and CSF identification. Slice 2 is shown in Figure 3(D), its histogram and triangle based analysis are shown in Figure 3(E and F). The triangles drawn in the Figure 3(C) and Figure 3(F) give the approximation of region overlapping. By ignoring the background, peaks show the presence of objects (GM, WM) significantly and next to them is the CSF. CSF lies in the closer region and sequential variation of gray levels. For more detail, Figure 4(A) shows the histogram of Figure 3(B) and Figure 4(B) represents the lines drawn around the peaks for probabilistic histogram. Figure 4(C) gives the probabilistic histogram and Figure 4(D) split the regions of interest (RIO) at a point to appropriately select the centroids. In probabilistic histogram gray levels are along X-axis and frequency along Y-axis, the highest peaks within the regions (GM, WM and CSF (Figure 4(D))) could be the best choice for centroids to precisely calculate the regions. The GM is within the region from 45 to 85 gray levels, WM from 86 to 140 gray levels while CSF lies from 141 up to 255. Figure 5 depicts the successful centroids within the specified ranges calculated with the proposed auto centroid selection method. These ranges have been evaluated from a large set of 18 subjects using T2 brain MR images, here is shown only two for description.

The steps involved for centriod selection method used after modification from 2 dimensional space to 3 dimensional space (Eq. (11–13)) [23] are:

### Steps

1. Filter the histogram 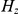 by selecting the peaks using the criterion mentioned hereunder
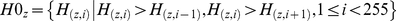
(5)
2. Calculate the set of peak points 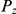 from the filtered histogram 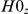 ignoring the unnecessary points that are less than 5% of the 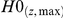, where 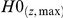 is the highest peak in the histogram 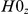.
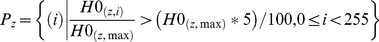
(6)
3. Calculate a new set of peak points 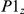 by measuring the distance between two peaks points 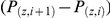 yields the significant representative points for each region.
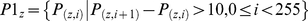
(7)The criterion in Eqs. (6 & 7) is proposed after testing on a set of T2 brain MR images (as shown in Table 1) for significant centroid calculation.
4. Now compute the candidate cluster centroids for each region using the following equation considering the range of the regions discussed previously.
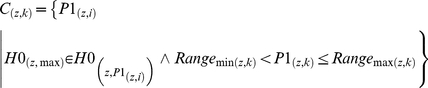
(8)Here 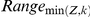 is the lower and 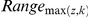 is the higher part of range for each image plane *z* to calculate number of centroids *k*. The selected centroids for each image plane are denoted by 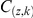.

Now the segmentation process is applied using identified centriods *C_Z_* and classified each voxel *V* into its identical cluster using the minimization of an objective function described in Eq. (3).

## Results and Discussion

**Figure 6:**
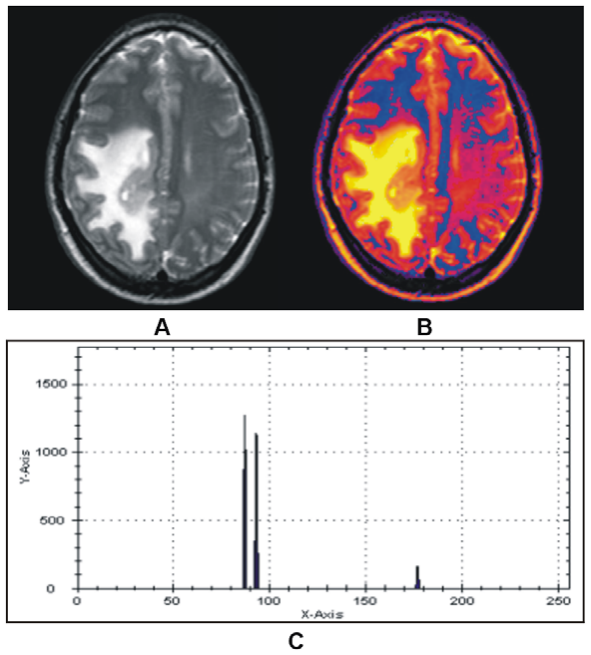
Color representation of T2 brain MR image with our proposed colorization method. (A) Abnormal T2 brain MR image of patient aged 32. (B) Color transformed image. (C) Selected centroids with proposed method.

**Figure 7:**
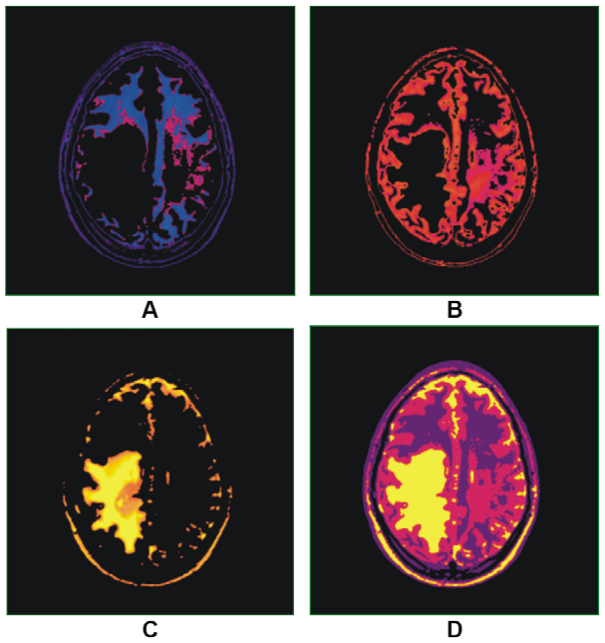
Results of hard and soft segmentation with our proposed method. (A) Gray matter. (B) White matter. (C) Cerebrospinal fluid with abnormality. (D) hard segmentation.

The colorized method has been tested by applying the proposed auto selection of centriod as shown in Figure 6. The T2 brain MR image in Figure 6(A) which shows the abnormality in the one half side of the image but the other side seems normal with proper GM, WM and CSF, while color representation of image in Figure 6(B) clearly differentiate these regions. To see the effect of the abnormality with selected centroids by using the proposed method are shown in Figure 6(C). Results after applying the segmentation method on the image in Figure 6(A) using the selected centroids are shown in Figure 7. Figure 7(A)–(C) gives soft segmentation results for GM, WM and CSF respectively. In Figure 7(C) the CSF clearly defines the abnormality. The hard segmentation is shown in Figure 7(D) where GM, WM and CSF are represented by purple, dark pink and yellow color respectively. Hence, Soft segmentation has been verified strongly in Figure 7(A)–(C). The more opaque pixels are classified into the extracted GM shown in Figure 7(A), the abnormal region on the one half side and the effected region on the other half side are not classified into GM proves the robustness of proposed segmentation method.

The colorization method label's different regions with different colors depend upon the density of the tissues to alleviate the visual perception so that the major regions are identified accurately with extra density measure. The separate representation of basic tissues in 3D space facilitates the volume estimation and other radiological evaluations. Colorized hard and soft segmentation techniques would help for better analysis and accurate clinical decisions. The advantage of soft segmentation is partial volume calculation which is crucial in T2 brain MR image study [9], [48]. Soft segmentation is also beneficial for volume estimation of identified tissues in 3D space on successfully removal of the skull. Changes in molecular movement and nuclear communication in T2 brain effects the translational and rotational relaxation. The possible reasons for these changes are different emotions, physical and psychiatric factors, gender, age, drugs or medication and other environmental effects [49]. The changes caused by these factors can be easily studied by careful analysis and comparison of soft segmented T2 brain MR tissues of normal and patients.

**Figure 8:**
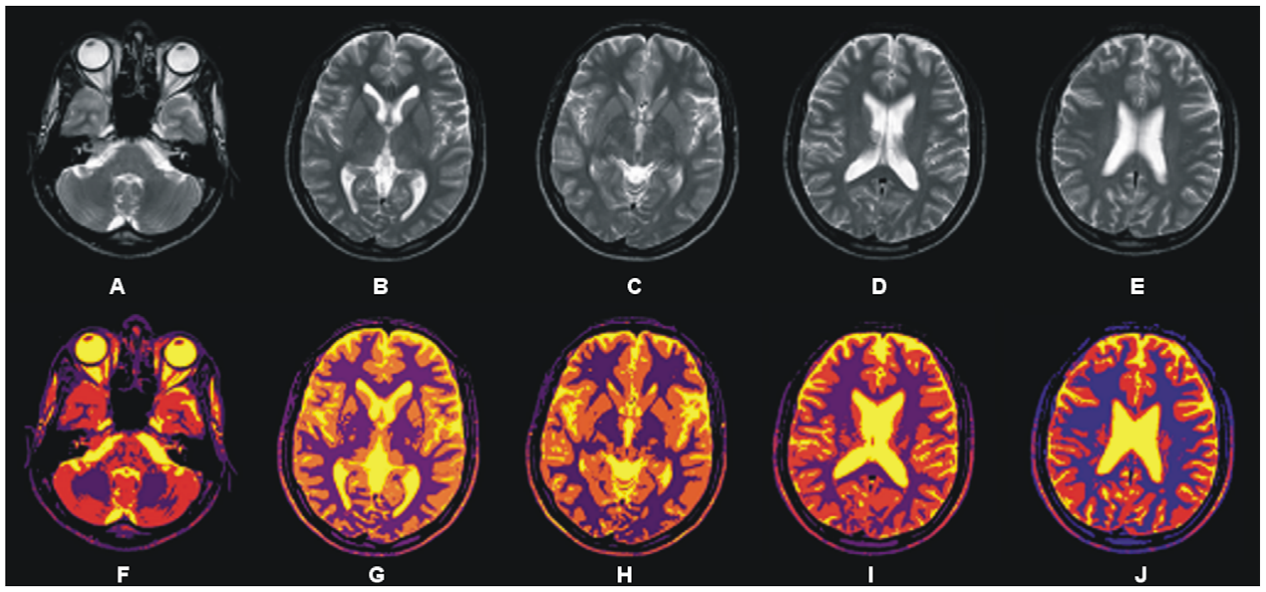
Showing results of 3D processing of proposed segmentation method. (A)–(E) Selected transaxial slices of T2 brain MR data set. (F)–(J) Colorized soft segmentation of slices (A)–(E).

Figure 8 depicts the segmentation results achieved by the proposed segmentation method for selected transaxial slice from T2 brain MR image data set. T2 brain MR image slices are shown in Figure 8(A)–(E) and segmentation results are shown in Figure 8(F)–(J).

**Figure 9:**
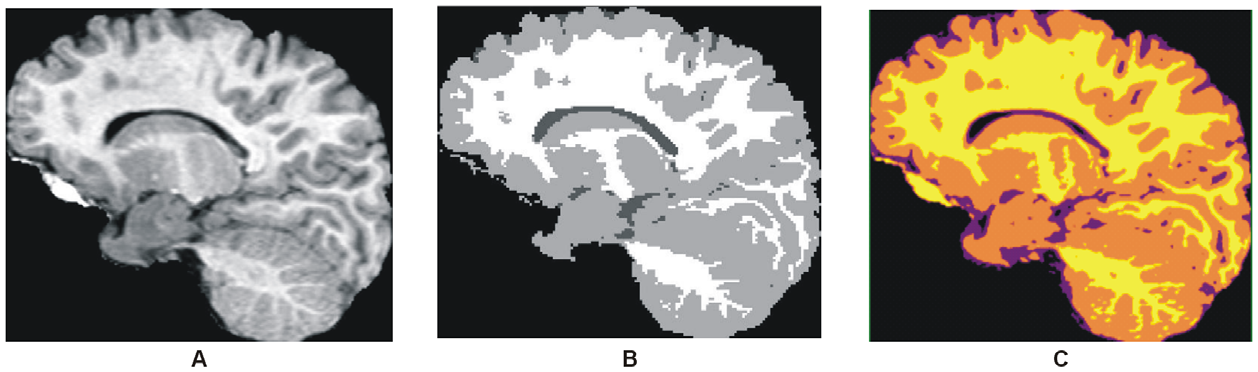
Comparison among the results obtained with our proposed segmentation method and EM-based segmentation. (A) T1 brain MR image of 2 year old child. (B) Segmented image with EM segmentation method. (C) Segmentation by proposed method.

Murgasova et al. [9] use population specific atlases for registration based and expectation maximization (EM) -based segmentation of T1 brain MRI in young children. Here we use the image of [9] for comparison purpose shown in Figure 9(A). Figure 9(B) show the results obtained by the EM-based segmentation method. Our segmentation method provides more precise result comparatively to the result shown in Figure 9(B). Our segmentation method in Figure 9(C) has the strong capability to represent the GM in orange color, WM in yellow and CSF in purple. The result revealed the significance of proposed method with much precise classification of WM and CSF and also satisfied that the proposed method works well with the T1 brain MR image of any age group or there is no need of specific population based atlases.

**Figure 10:**
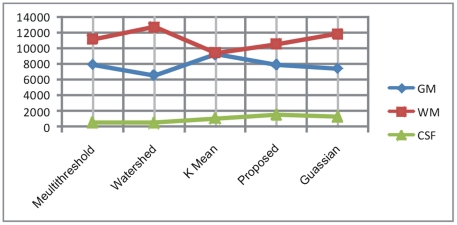
Showing the volumetric chart for comparison of proposed method with other segmentation methods.

In Table 2 the comparison is made between the proposed and three other segmentation methods using Gaussian clustering as a standard method with conformity of two radiologists. The comparison is made using Jaccard coefficient on average volume (in pixels) in 2D space. The segmentation results are obtained using multithreshold, watershed, K-mean, Gaussian and proposed method on total 12 T2W axial selected slices (four numbers of selected slices from three subjects each). The results from Gaussian clustering are obtained using Analyze 10.0 [50] by the consultation of two radiologists. The region wise Jaccard coefficient is calculated amongst the volume of extracted regions obtained from other segmentation methods and the volume of extracted regions obtained using standard Gaussian method. Volume in pixels and Jaccard coefficient shows that the segmentation results with proposed method are closer to the results of standard method (Gaussian) as compared to the other segmentation methods. Volume of GM, WM and CSF is also represented in a chart in Figure 10. It is easily deducible by observing the Table 2 and volume based chart in Figure 10 that the results produced with our proposed segmentation method are very similar to the standard segmentation method adopted here for comparison purpose that proves the robustness of our proposed segmentation method. The segmentation results produced by other methods vary in segmentation of different anatomical regions. The Jaccard coefficient (*Jc*) is calculated using the Eq (9).
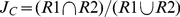
(9)Where R1 and R2 are the same regions extracted from other segmentation methods and Gaussian respectively.

**Table 2:** Comparison and Jaccard Coefficient of GM, WM and CSF in pixels (Avg).

Algorithm		Volume in pixels (Avg)	Jaccard Coefficient
**Multi Threshold**	GM	7973	0.03283891
	WM	11220	0.0305858
	CSF	573	0.41649695
**Watershed**	GM	6596	0.06186887
	WM	12763	0.03381799
	CSF	521	0.45502092
**K Mean**	GM	9263	0.10741826
	WM	9457	0.11554828
	CSF	1097	0.1181672
**Proposed**	GM	7921	0.02957042
	WM	10567	0.06050233
	CSF	1546	0.05277494
**Gaussian clustering (as standard method)**	GM	7466	-
	WM	11928	-
	CSF	1391	-

**Figure 11:**
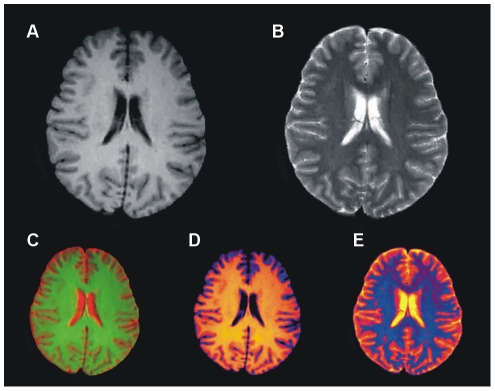
Comparison among the colorized results of our proposed method and MCMxxxVI [**35**]. (A) T1 image. (B) T2 image. (C) The fused color image is obtained with MCMxxxVI. (D)–(E) Representing the color version of images (A)–(B) respectively, produced by our proposed method.

The color image shown in Figure 11(C) is obtained using the method adopted by MCMxxxVI [35], where red color is assigned to T2 image shown in Figure 11(B) and green is assigned to T1 image shown in Figure 11(A) and then these images are fused to get color image. In this colorization process the boundaries of GM and WM are fused, making the difference unperceivable. The internal variations shown in GM of T2 image in Figure 11(B) are also not visible in Figure 11(C). Figure 11(D)–(E) are the colorized versions (obtained with the proposed colorization method) of image in Figure 11(A)–(B). The anatomical regions are easily identifiable along with the internal variations in the colorized T1 and T2 images shown in Figure 11(D)–(E) respectively which proves the robustness of our proposed colorization method.

It is visually verifiable that the proposed colorization method effectively colorizes each anatomical region for each MR image sequence. Since various pathological evaluations requires the different multi-parametric sequences for exact analysis [51] consequently it requires that each sequence should be colorized to enhance visual probing and evaluation. The proposed method colorizes each image sequence independently to improve further pathological evaluation and enhances visual probing.

**Figure 12:**
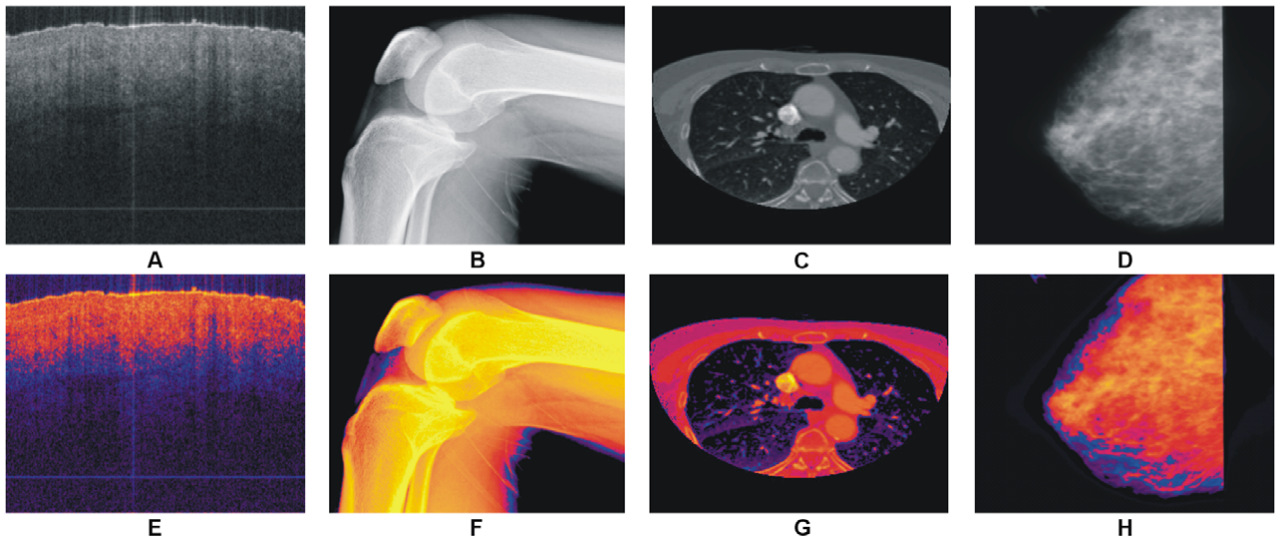
Results of our proposed colorization method with other medical imaging modalities. (A) OCT image of nail under skin. (B) Digital X-Ray of knee. (C) CT image of thorax. (D) Mammographic image. (E)–(H) Colorized images of (A)–(D) with our proposed method respectively.

In Figure 12 some images from other imaging modalities are shown along with their color version processed with the proposed colorization method. It has been reported in research that single MRI sequence (T1, T2 and etc) is not sufficient enough for precise pathological evaluation in most of the cases [51]. Our proposed method colorizes each image sequence independently that are helpful in further pathological study and analysis.

Here in Table 3 processing time based comparison is shown between the proposed and other segmentation methods. Based on this comparison it is verified that our proposed method is significantly robust comparatively to the other segmentation methods. The average processing time is calculated on a set of 10 selected images. The average processing time for colorization is demonstrated in Table 4 where the time is calculated in milliseconds separately for a single slice and also for a set of 20 images. All the experiments are performed using Dell Latitude 610 1.79 GHz laptop with 1 GB of RAM and 1024 MB paging file size.

**Table 3:** Showing the computation time to segment objects from one slide by different algorithms.

Algorithm	Dimension	Number of Clusters	Iterations Required	Computation Time (Avg) millisecond
**Multi Threshold**	256×256	4	1	1.709
**Watershed**	256×256	-	1	2.894
**K Mean**	256×256	4	31	47.852
**Proposed**	256×256	4	1	0.142

**Table 4:** Showing the computation time to colorize gray brain MRI images of different dimensions and imaging modalities.

Image Type	Slice Dimension	Time taken to colorized inMillisecond (Avg)	Time taken to colorized 20 slices in millisecond (Avg)
**MRI(T2)**	256×256	1.653	32.74
	512×512	6.968	143.92
**MRI(T1)**	256×256	1.631	32.94
	512×512	7.015	139.83
**MRI(FLAIR)**	256×256	1.698	33.12
	512×512	6.892	136.74
**CT**	256×256	1.538	-
**OCT**	256×256	1.329	-

**Figure 13:**
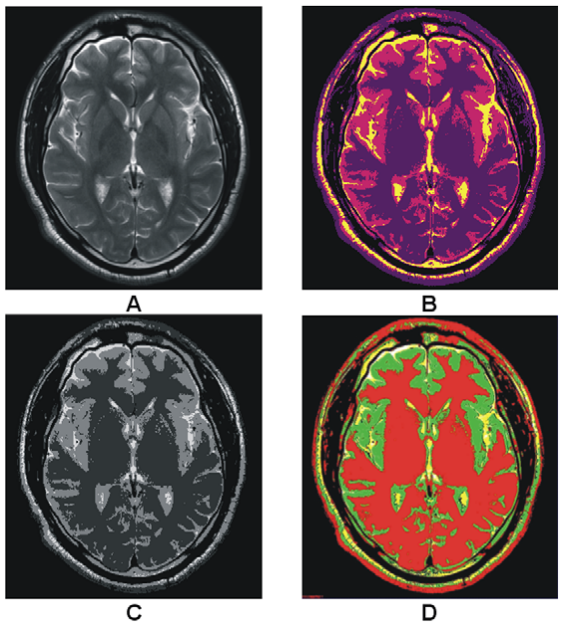
Comparison among the results obtained with our proposed segmentation method and other standard segmentation methods using the image obtained with Siemens MRI scanner. (A) T2 brain MRI image with Siemens MAGNETOM Aera 1.5 T [52] with dimension 600×600. (B) Segmentation with proposed method. (C) Watershed segmentation. (D) Gaussian classifier segmentation using Analyze 10 [50].

For the verification of the proposed method's working with the brain MRI images scanned through other vendors of MRI scanners, we use the T2 image acquired with Siemens MAGNATON Aera 1.5 T [52] shown in Figure 13. The comparison of segmentation is made using watershed and Gaussian clustering with the proposed method. For visual verification the results of segmentation are shown in Figure 13 and Table 5 shows the volumetric measurement and Jaccard coefficient for comparison purpose. This result provides the evidence of successful implementation of our proposed segmentation method on the MR images acquired with some other vendor's MRI scanner.

**Table 5:** Showing the comparison of volume and Jaccard coefficient of proposed and watershed with standard Gaussian segmentation methods of the image shown in Figure 4.

Algorithm		Volume in pixels (Avg)	Jaccard Coefficient
**Watershed**	GM	117228	0.04089931
	WM	69950	0.13315136
	CSF	9381	0.23194695
**Proposed**	GM	125290	0.00766684
	WM	65719	0.10239034
	CSF	23229	0.21376319
**Gaussian**	GM	127226	-
	WM	53511	-
	CSF	15047	-

Bauer CM et al. [53] conducted a study to examine that the dual echo pulse sequence is subjected to the vendor based variance by using three different vendor's MRI scanners (Siemens, General Electric and Philips). They examined the peaks positions and the width variance in histogram of different MR images acquired with different scanner vendors. Furthermore similar study can be established to find the variance amongst the results of different scanner vendors and then incorporate these findings in histogram analysis to make the proposed method more robust and platform independent. This work is included in our future aim.

**Figure 14:**
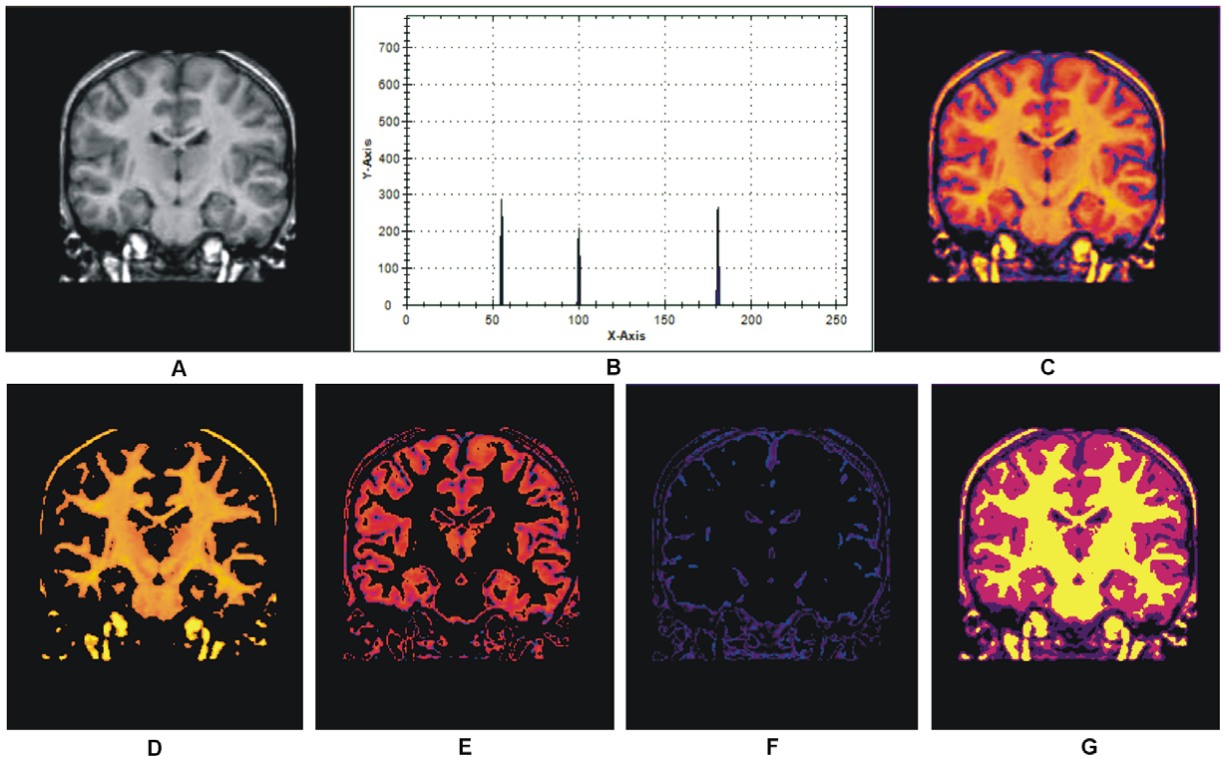
Results of our proposed colorization and segmentation method on T1 brain MR image. (A)T1 Brain MRI image [54]. (B) Selected centroids with the proposed auto centroid selection method. (C) Color transformed image of (A) with proposed colorization method. (D)–(E) Extracted matter one, extracted matter second and CSF respectively with proposed segmentation method utilizing the selected centroids shown in (B). (G) Whole segmented image with proposed method.

**Figure 15:**
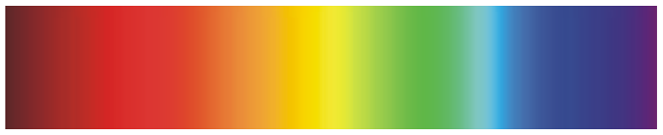
Color Image from which the 200 color derived (courtesy of [44]).

The research is purely conducted for T2 brain MR images, but it also works well with T1, Proton Density (PD) and FLAIR brain MR images. The results are included here for visual verification in Figure 14 for T1 MR image and is obtained from the IBSR data set [54]. The segmentation results shown in Figure 9 and Figure 14 verify the successful implementation of proposed segmentation method for T1 brain MR images. Figure 15 shows the image from which the colors are derived to utilize in our aforementioned colorization method.

### Conclusion

In this research work we implement two methods. The first is colorization method to colorize the gray scale MR images to enhance the visual perception and increase discrimination. The proposed methods generate appreciative results on applying to the T2 brain MR images. The results generated with the colorization method are excellently refined and clearly unveil the hidden information that is difficult to observe with naked eye from the gray scale image. The variation in the tissue density or opaqueness with in a specific region or in the whole image can be easily studied. The proposed colorization method makes it so flexible to directly integrate with the image digitization process after a bit enhancement or modifications to acquire color images along with gray images. Second is an additional clustering method guided with auto selected centroids. The segmentation results successfully discriminate the regions with different density measure and label each distinct region with a different color. The results for the proposed segmentation method were testified by comparing the results with the EM-based segmentation method, FCM and k-mean method. The proposed additive clustering method provides appreciative results comparatively to the methods mentioned above. On the other hand soft segmentation appreciatively segments all guided regions. The purpose of the study was segmentation of T2 brain MR images however it works well where there is sufficient contrast among the different regions in T1, PD and FLAIR images.
